# A Case of Cryptococcal Meningitis in an Immunocompetent Individual

**DOI:** 10.7759/cureus.99307

**Published:** 2025-12-15

**Authors:** Elodie Baumgartner, Lexi Weltin, John J Farrell

**Affiliations:** 1 Infectious Disease, University of Illinois College of Medicine Peoria, Peoria, USA; 2 Pediatrics and Child Health, University of Illinois College of Medicine Peoria, Peoria, USA; 3 Microbiology and Immunology, OSF System Lab, Peoria, USA

**Keywords:** cryptococcal meningitis, immunocompetent cryptococcal meningitis, mortality in cryptococcal meningitis, mri mimics of cva, non-hiv cryptococcal meningitis

## Abstract

Cryptococcal meningitis is classically associated with immunocompromised hosts but can also occur in individuals without identifiable immune dysfunction. Its nonspecific and often indolent presentation may delay diagnosis, particularly when early neuroimaging suggests alternative etiologies. We report a case of a 49-year-old immunocompetent man who presented with progressive headaches, sensory changes such as transient hearing loss, visual changes, and balance issues. Initial imaging showed a hyperdense area on CT, which was interpreted as a possible cerebrovascular accident, delaying recognition of an infectious process. Cerebrospinal fluid (CSF) evaluation ultimately confirmed cryptococcal meningitis, and the patient improved with targeted antifungal therapy and intracranial pressure (ICP)-directed management. This case highlights diagnostic challenges in immunocompetent hosts and underscores the importance of maintaining suspicion for fungal meningitis even without classic risk factors.

## Introduction

*Cryptococcus neoformans* and *C. gattii* are true yeast that possess a prominent polysaccharide capsule that stains pink/red with mucicarmine but is impermeable to India ink and behave as opportunistic pathogens [1]. *C. neoformans* is associated with meningitis in immunocompromised individuals, particularly those with an HIV infection who have progressed to AIDS with a CD4 count below 100 cell/mL [2,3]. However, emerging evidence suggests that *C. neoformans* pneumonia and meningitis are occurring in immunocompetent patients more frequently [4,5]. Factors contributing to the rise in incidence of *C. neoformans* infections in immunocompetent individuals may include increasing human lifespans as well as expanding use of immune modulators in medical practice; but 20% of cases appear to be immune competent, some of whom were ultimately diagnosed with leukocyte adhesion deficiency (LAD) [5]. Cryptococcal meningitis is extremely difficult to treat due to the severe neurological sequelae, raised intracranial pressure (ICP), and immune reconstitution inflammatory syndrome (IRIS) [6].

The typical clinical presentation includes fever, headache, and photophobia, but classic signs associated with bacterial meningitis such as nausea and vomiting, and nuchal rigidity may not be present. Atypical manifestations such as acute cerebellar signs have been reported [3]. Neuroimaging usually does not inform the diagnostic workup. Although subtle MRI findings including leptomeningeal enhancement or dilated perivascular spaces may be present, CT imaging of the head is typically normal and findings of miliary nodules or frank ventriculitis are extremely rare [3]. Definitive diagnosis relies on cerebrospinal fluid (CSF) analysis, with CSF culture being the gold standard due to their high sensitivity and specificity [4]. Also, multiplex PCR panels (mPCR) of CSF are known to produce false negatives. The sensitivity of mPCR panels is significantly lower in patients with a low CSF yeast burden (e.g., <100 CFU/mL) [4].

This case report highlights cryptococcal meningitis in an immunocompetent patient, emphasizing its diagnostic challenges and the importance of maintaining a high index of suspicion even in the absence of classic risk factors.

## Case presentation

**Figure 1:**
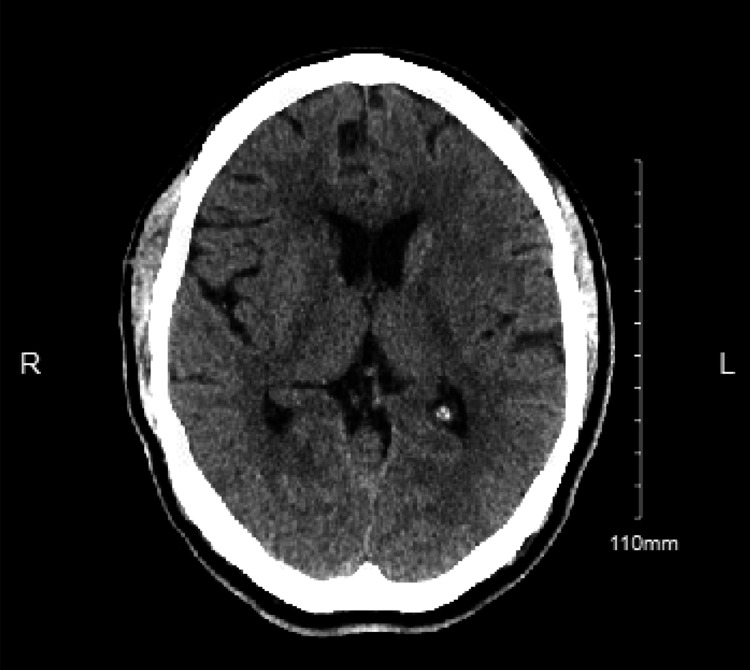
Day 0 CT of head w/o contrast. Abnormal FLAIR signal in the right caudate and left basal ganglia, with corresponding diffusion restriction in the right caudate and bilateral corona radiata/centrum semiovale white matter. Some T2 shine through is noted involving the right caudate. FLAIR: Fluid-attenuated inversion recovery

A 49-year-old man with no past medical history and a family history of diabetes mellitus initially presented to the emergency department with three days of a throbbing frontal/occipital headache with associated nausea, vomiting, flashing lights, vertigo, and numbness and tingling of the left hand, left leg, and right face. At baseline, he works with heavy machinery and as a farmhand. At the initial presentation, CT of head/brain without contrast was ordered and revealed faint areas of hypodensity in the right head of the caudate nucleus, which were concerning lacunar infarcts (Figure 1). CT angiography of head was then ordered and revealed no large vessel occlusion. Subsequent MRI of brain demonstrated abnormal diffusion restriction of the bilateral corona radiata/centrum semiovale white matter and right caudate. Diffuse mild effusion and T2/fluid-attenuated inversion recovery (FLAIR) signal abnormality were also noted throughout the cerebellum. The patient was subsequently diagnosed with a cerebrovascular accident and was discharged with 300 mg aspirin daily and 50-325-40 mg Fioricet every four hours as needed. 

**Figure 2:**
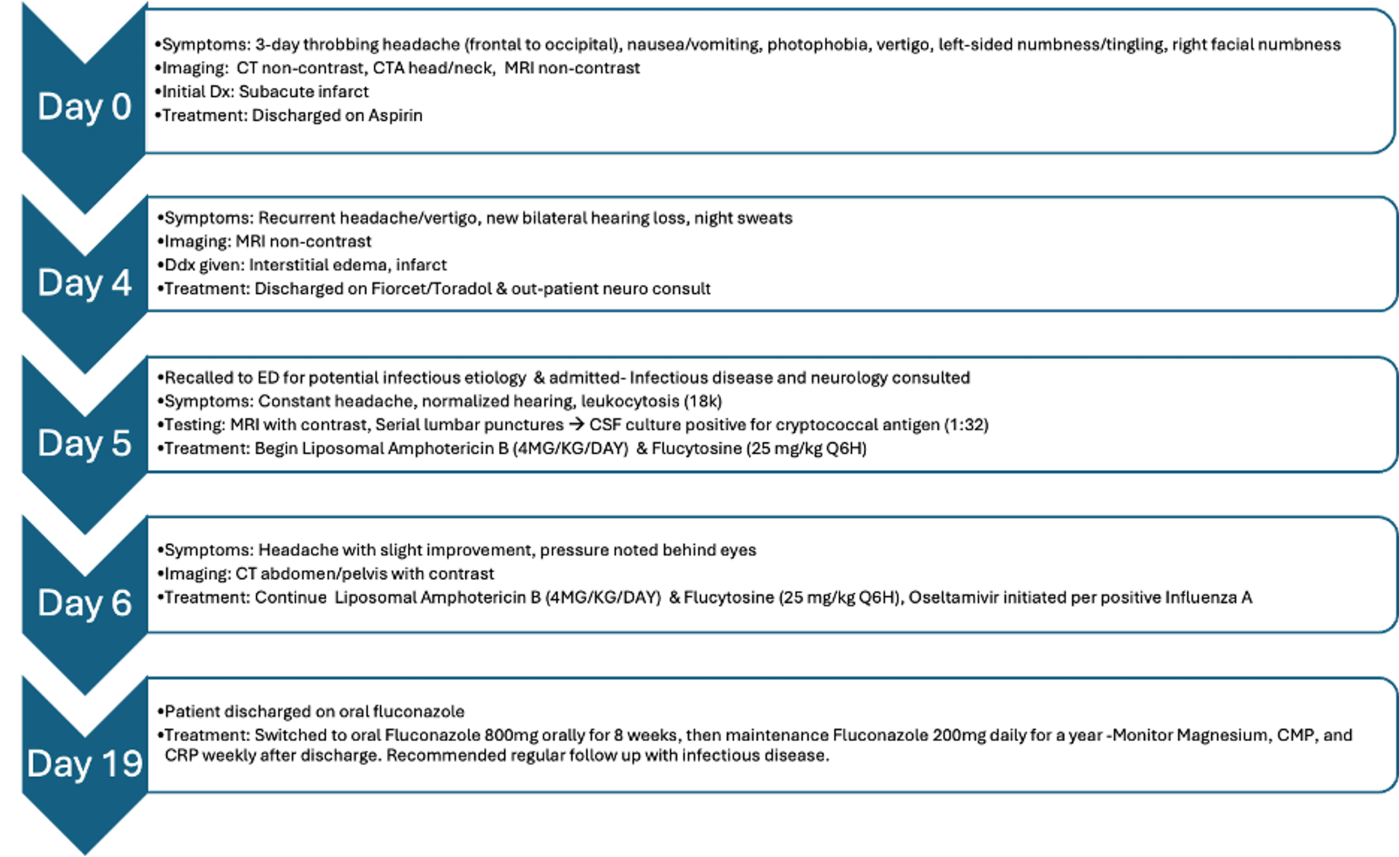
Abbreviated timeline of events This illustrates the main series of events leading to the diagnosis of cryptococcal meningitis. It highlights the most pertinent symptoms, labs/imaging, and treatments associated with this case.

Four days later, the patient returned to the emergency department with the same symptomatic headache and stated he had new onset bilateral hearing loss and no relief after taking the previously prescribed medications as needed (Figure 2). At this time, he was unsure of fevers but endorsed night sweats and demonstrated leukocytosis with an elevated white blood cell count on complete blood count (CBC) which was attributed to the inflammatory process of his stroke at the time (Table 1). Infectious etiology was low on the differential due to the patient not manifesting tachycardia or altered mental status. MRI did not indicate a stroke, but Neurology consult felt that continuing with secondary treatment for stroke was appropriate and that the hearing loss could be contributed to a potential past stroke.

**Table 1:** Day 4 CBC with auto differential WBC, absolute neutrophils, and neutrophils were elevated. Lymphocytes were low. CBC: Complete blood count; WBC: White blood cell; RBC: Red blood cell; HGB: Hemoglobin; HCT: Hematocrit; MCV: Mean corpuscular volume; MCH: Mean corpuscular hemoglobin; MCHC: Mean corpuscular hemoglobin concentration; RDW: Red cell distribution width; MPV: Mean platelet volume; NRBC: Nucleated red blood cell

Test	Result	Reference Range
WBC	14.47 (High)	4.00-12.00 x 10^3^/mcL
RBC	5.46	4.40-5.80 x 10^6^/mcL
HGB	16.1	13.0-16.5 g/dL
HCT	46.7	38.0-50.0%
MCV	85.5	82.0-96.0 fL
MCH	29.5	26.0-32.0 pg
MCHC	34.5	31.0-36.0 g/dL
Platelet count	349	140-440 x 10^3^/mcL
RDW	13.4	11.8-15.5 %
MPV	9.5	8.0-12.6 fL
Neutrophils	82.3 High	40.0-68.0%
Lymphocytes	12.0 Low	19.0-49.0%
Monocytes	4.6	3.0-13.0%
Eosinophils	0.5	0.0-8.0%
Basophils	0.6	0.0-1.0%
Absolute neutrophils	11.91 (High)	1.40-5.30 x 10^3^/mcL
Absolute lymphocytes	1.74	0.90-3.30 x 10^3^/mcL
Absolute monocytes	0.67	0.10-0.90 x 10^3^/mcL
Absolute eosinophils	0.07	0.00-0.50 x 10^3^/mcL
Absolute basophils	0.08	0.00-0.10 x 10^3^/mcL
NRBC per 100 WBC	0	NA

**Figure 3:**
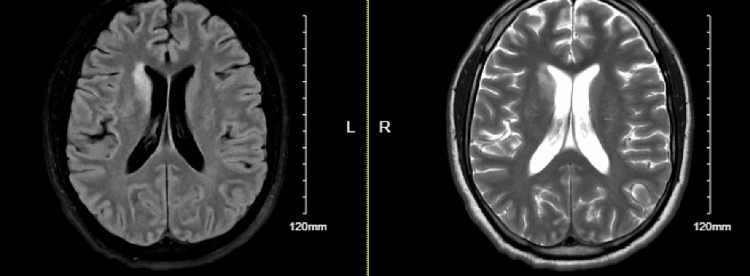
Day 4 MRI non-contrast Radiologic interpretation of increased hyperintensities in both ADC and DWI along the bilateral corona radiata right anterior caudate nucleus, dorsal left putamen and bilateral cerebellar hemispheres with an exception of a small area in the caudate. These imaging findings are consistent with acute or subacute interstitial disease. Differential includes inflammatory/post inflammatory, infectious/postinfectious, autoimmune, and PRES or demyelinating processes. DWI: Diffusion-weighted imaging; ADC: Apparent diffusion coefficient; PRES: Posterior reversible encephalopathy syndrome

A second MRI of the brain was ordered and was significant for an increased hyperintensity along the bilateral corona radiata right anterior caudate nucleus, dorsal left putamen, and bilateral cerebellar hemispheres (Figure 3). The increased signal on diffusion-weighted imaging (DWI) and apparent diffusion coefficient (ADC) maps indicate there is acute or subacute interstitial edema not caused by acute infarct. The patient experiencing headaches as well as the sensory deficits indicate elevated ICP, which can result in cerebral interstitial edema. Patient felt better on Toradol and was discharged at this time with Fioricet and Toradol for 2-4 days. Upon later review of the MRI by a new neurologist on shift, infectious etiology became a concern and the patient was recommended to get a lumbar puncture.

The patient arrived at the tertiary care emergency department two days later and reported that the headache was still constant with relief only provided by ketorolac and that his hearing loss and numbness was only present when the headache was present. He also reports potential weight loss but is unsure of the amount of weight loss. He weighed 170 lbs at admission and 170 lbs a year prior. He denied recent travel outside of the United States and denied any sick contacts, shortness of breath, hemoptysis, dysuria, diarrhea, seizure history, or traumatic brain injury. At this time, the patient was ultimately admitted to the hospital for lumbar puncture and further work-up. On admission, the patient was hemodynamically stable with an elevated blood pressure of 141/98. CBC with differential demonstrated continued leukocytosis (Table 2). On physical examination, he demonstrated mild neck tenderness to palpation with an otherwise unremarkable exam including no focal neurological deficits. 

**Table 2:** Day 5 CBC with auto differential. WBC, neutrophils, absolute neutrophils, and monocytes were elevated. Lymphocytes were low. CBC: Complete blood count; WBC: White blood cell; RBC: Red blood cell; HGB: Hemoglobin; HCT: Hematocrit; MCV: Mean corpuscular volume; MCH: Mean corpuscular hemoglobin; MCHC: Mean corpuscular hemoglobin concentration; RDW: Red cell distribution width; MPV: Mean platelet volume; NRBC: Nucleated red blood cell

Test	Result	Reference Range
WBC	18.01 (High)	4.00-12.00 x 10^3^/mcL
RBC	5.17	4.40-5.80 x 10^6^/mcL
HGB	15.4	13.0-16.5 g/dL
HCT	44.9	38.0-50.0%
MCV	86.8	82.0-96.0 fL
MCH	29.8	26.0-32.0 pg
MCHC	34.3	31.0-36.0 g/dL
Platelet count	329	140-440 x 10^3^/mcL
RDW	13.8	11.8-15.5%
MPV	10.0	8.0-12.6 fL
Neutrophils	75.6 (High)	40.0-68.0%
Lymphocytes	18.0 (Low)	19.0-49.0%
Monocytes	5.7	3.0-13.0%
Eosinophils	0.3	0.0-8.0%
Basophils	0.4	0.0-1.0%
Absolute neutrophils	13.61 (High)	1.40-5.30 x 10^3^/mcL
Absolute lymphocytes	3.25	0.90-3.30 x 10^3^/mcL
Absolute monocytes	1.02 (High)	0.10-0.90 x 10^3^/mcL
Absolute eosinophils	0.06	0.00-0.50 x 10^3^/mcL
Absolute basophils	0.07	0.00-0.10 x 10^3^/mcL
NRBC per 100 WBC	0	NA

A full workup was ordered, and Infectious Disease was consulted for the patient to determine any potential infectious causes of the patient’s persistent headaches. Lumbar puncture CSF studies revealed a low glucose of 38 mg/dL, high protein of 70 mg/dL, high total nucleated cell counts of 280 cm^3^, red blood cell (RBC) count of 116 cm^3^, 89% lymphocytes, 6% monocytes, 5% neutrophils, and an opening pressure of 29 cm H_2_O (Table 3). Other pertinent negative tests performed during the infectious workup include Negative West Nile virus antibody IgG and IgM, Lyme IgG with antibody reflex, CSF herpes simplex 1 and 2, CSF venereal disease, and enterovirus polymerase chain reaction (PCR) (Table 4). 

**Table 3:** CSF analysis and profile upon admission CSF analysis indicated a diagnosis of cryptococcal meningitis. CSF: Cerebrospinal fluid; RBC: Red blood cell; TNC: Tenascin-C; VDRL: Venereal Disease Research Laboratory test

CSF Analysis		
CSF culture	Cryptococcus neoformans	No growth (Sterile)
CSF glucose	38 mg/dL	40-70 mg/dL (~2/3 of serum glucose)
CSF protein	70 mg/dL	15-45 mg/dL (lumbar)
RBC count	116/mm³	0 cells/µL (unless traumatic tap)
TNC count	280/mm³	0-5 cells/µL (adults)
Differential: Neutrophils	5%	0-6% (adults)
Differential: Lymphocytes	89%	40-80% (adults)
Differential: Monocytes	6%	5-45% (adults)
CSF lactic acid	3.9 mmol/L	10-22 mg/dL (1.1-2.4 mmol/L)
CSF VDRL	Negative	Non-reactive (Negative)
CSF cryptococcus antigen qual.	Positive	Negative
CSF cryptococcus antigen quant.	1:32 → 1:64	<1:8 titer
Antimicrobial susceptibility (Yeast)	Cryptococcus neoformans	Sensitive/Intermediate/Resistant
Histoplasma/Blastomyces antigen	Not detected	Negative
CSF IgG Profile		
CSF IgG index	1.34	0.29-0.59
CSF IgG	13.6 mg/dL	0.8-7.7 mg/dL
CSF albumin	35.2 mg/dL	8-32 mg/dL
CSF IgG/albumin	0.39	≤0.27
CSF IgG synthesis rate	43.56 mg/24h	≤3.3 mg/day
Serum IgG	1,380	700-1,600 mg/dL
IgG/albumin (Serum)	0.29	0.2-0.4
Albumin quotient (CSF/Serum)	7.33	≤0.007
Serum Albumin	4,800	3.5-5.0 g/dL

**Table 4:** Infectious disease and inflammatory markers upon admission Notable pertinent negatives were present in the infectious agent's section. Inflammatory markers and elevated WBC align with infectious etiology. PCR: Polymerase chain reaction; CSF: Cerebrospinal fluid; CNS: Central nervous system; CMV: Cytomegalovirus; TB: Tuberculosis; ESR: Erythrocyte sedimentation rate; WBC: White blood cell; ANC: Absolute neutrophil count; ANA: Antinuclear antibody; ANCA: Antineutrophil cytoplasmic antibody; IFA: Indirect immunofluorescence assay; DHR: Dihydrorhodamine; CGD: Chronic granulomatous disease

Infectious Disease Testing		
Test	Result	Reference Range
West Nile Virus IgG & IgM	Negative	Negative (Non-reactive)
Herpes Simplex 1 & 2 PCR (CSF)	Negative	Negative (Non-reactive)
Lyme CNS infection IgG	Negative	Negative (Non-reactive)
Enterovirus PCR (CSF)	Negative	Enterovirus PCR (CSF): Negative (Not detected)
Lyme antibody	0.13	Lyme antibody: Negative (Non-reactive)
CMV IgG & IgM	Normal	Negative or positive (past infection)
Toxoplasma IgM	<3.0 (Normal)	Negative (Non-reactive)
Hep B surface antibody/core Ab	Negative	≥10 mIU/mL (Protective)
Hep C antibody	Negative	Negative (Non-reactive)
Syphilis IgG/IgM	Negative	Negative (Non-reactive)
Quantiferon TB Gold	Normal	Negative (Non-reactive)
Hematologic/Inflammatory Markers		
ESR	12 mm/h	0-20 mm/hr (varies by age/sex)
CRP	<0.10 mg/dL	<1.0 mg/dL (Low risk)
CBC: WBC	18.01 x 10³/mcL	4,000-11,000 cells/µL
CBC: ANC	13.61 x 10³/mcL	1,500-8,000 cells/µL
ANA screen	Negative titer	Negative (<1:40 titer)
ANCA IFA screen	Normal <1:20	Negative
DHR flow cytometry (ANC)	19,680 cells/mcL *Not consistent with CGD	≥90% of control

**Table 5:** CMP panel upon admission These were notable for elevated ALT and glucose. CMP: Comprehensive metabolic panel; ALT: Alanine aminotransferase; BUN: Blood urea nitrogen; A/G: Albumin/globulin; AST: Aspartate aminotransferase; SGOT: Serum glutamic-oxaloacetic transaminase; SGPT: Serum glutamic-pyruvic transaminase; GFR: Glomerular filtration rate

CMP Component	Value	Reference Range
Sodium	137 mmol/L	136-145 mmol/L
Potassium	4.4 mmol/L	3.5-5.1 mmol/L
Chloride	102 mmol/L	98-107 mmol/L
CO₂ (Venous)	24 mmol/L	22-30 mmol/L
Anion gap	11 mmol/L	<18 mmol/L
Glucose	130 mg/dL (High)	70-99 mg/dL
BUN	16 mg/dL	9-21 mg/dL
Creatinine	0.99 mg/dL	0.70-1.30 mg/dL
BUN/Creatinine ratio	16	12-20
Total protein	6.4 g/dL	6.0-8.0 g/dL
Albumin	3.6 g/dL	3.5-5.0 g/dL
A/G Ratio	1.3	1.0-2.2
Calcium	9.0 mg/dL	8.7-10.5 mg/dL
Total bilirubin	0.3 mg/dL	0.2-1.2 mg/dL
AST (SGOT)	33 U/L	6-42 U/L
ALT (SGPT)	85 U/L (High)	6-55 U/L
Alkaline phosphatase	70 U/L	40-150 U/L
Estimated GFR	>60	≥60

Ultimately, with both the CSF culture and cryptococcal antigen screen the sensitivity is 98%. The patient’s CSF culture demonstrated *Cryptococcus neoformans. *The* *patient also demonstrated a positive Cryptococcus antigen screen and antigen titer of 1:32, which further confirmed the source of headaches and associated symptoms. At this time, induction therapy was started with liposomal amphotericin B at 4 mg/kg once daily and flucytosine 25 mg/kg every six hours. In the setting of no documented and verbalized past medical history, the patient underwent further testing to identify potential immune deficiency given his active Cryptococcus infection (Table 6). However, the studies done to assess immunocompromise were found to be inconclusive. He was hepatitis B surface antibody and core total antibody negative, hepatitis C antibody negative, syphilis IgG/IgM negative, quantiferon tuberculosis (TB) gold testing within normal limits (WNL), cytomegalovirus (CMV) IgG and IgM WNL, toxoplasma IgM antibody WNL, human immunodeficiency PCR test negative, and had a CD4 cell count of 641 cells/mm^3^. The patient did demonstrate an elevated absolute neutrophil count (ANC) of 19,680 cells/microliter on dihydrorhodamine (DHR) flow cytometric blood tests, but other values were WNL, and the results were not consistent with chronic granulomatous disease (CGD) or myeloperoxidase deficiency. 

**Table 6:** Autoimmune/paraneoplastic evaluation serum panel Negative serum panel ruled out autoimmune and paraneoplastic causes. CBA: Cell-based assay; AGNA: Antiglial nuclear antibod; ANNA: Antineuronal nuclear antibody; CASPR-IgG: Contactin-associated protein-like 2 immunoglobulin G; GABA-B-R: Gamma-aminobutyric acid type B receptor; GAD65: Glutamic acid decarboxylase 65; GFAP: Glial fibrillary acidic protein; IFA: Indirect immunofluorescence assay;  MGLUR1: Metabotropic glutamate receptor 1; IgLON5: Immunoglobulin LON5; LGI1: Leucine-rich glioma-inactivated 1; NF: Neurofilament; NMDA-R: N-methyl-D-aspartate receptor; PCA: Purkinje cell antibody; TR: Thyroid receptor; PDEIS: Paraneoplastic encephalitis disease immunoscreen; PDE10A: Phosphodiesterase 10A; T46IS: T46 immunoscreen; TRIM46: Tripartite motif-containing 46

Test Name	Result
AMPA-R AB CBA	Negative
Amphiphysin AB S	Negative
AGNA 1 S	Negative
ANNA 1 S	Negative
ANNA 2 S	Negative
ANNA 3 S	Negative
CASPR-IgG CBA S	Negative
CRMP 5 IGG	Negative
DPPX AB CBA S	Negative
GABA-B-R AB CBA S	Negative
GAD65 AB assay S	0.02 (WNL)
GFAP IFA S	Negative
MGLUR1 AB IFA S	Negative
IgLON5 CBA S	Negative
LGI1-IGG CBA S	Negative
Neurochrongrin IFA S	Negative
NF IFA S	Negative
NMDA-R AB CBA S	Negative
PCA 1	Negative
PCA 2	Negative
PCA TR	Negative
PDEIS PDE10A AB IFA S	Negative
Septin-7 IFA S	Negative
T46IS TRIM46 AB IFA S	Negative

Thus, the patient did not have an identifiable source of immunocompromise despite having *Cryptococcus neoformans* induced meningitis. 

As the patient started induction therapy, he continued to demonstrate significantly debilitating headaches while admitted despite additional symptomatic treatment. It was subsequently determined at that time that the patient would potentially benefit from serial lumbar punctures to reduce ICP with a goal opening pressure of around 25 cm H_2_O with symptomatic improvement. The repeat lumbar puncture demonstrated an increased opening pressure at 36 cm H_2_O from the initial 29 cm H_2_O. A repeat MRI of the brain demonstrated similar patchy areas of cerebral and cerebellar T2 FLAIR hyperintensity with corresponding intraparenchymal and leptomeningeal enhancement. It did not demonstrate lesions that were classic for cryptococcoses. A few days later, another Cryptococcus antigen titer came back at a value of 1:64, demonstrating potential improvement from the induction therapy but still the presence of an active infection. The patient concurrently demonstrated an improvement in symptomatic headaches. A third lumbar puncture was obtained given the previous elevated opening pressures. This lumbar puncture demonstrated an opening pressure of 27 cm H_2_O, thus achieving its goal with symptomatic improvement from his headaches with full resolution. The patient ultimately completed induction therapy and was transitioned to fluconazole 800 mg by mouth daily for eight weeks and then scheduled for 200 mg of fluconazole by mouth daily for one year total duration. One month after admission, the patient received a repeat MRI of the brain that demonstrated an overall improvement in areas of T2 prolongation with minimal residual enhancement (Figure 3). This imaging was consistent with the patient’s resolution of symptomatic headaches and neck tenderness. 

**Figure 4:**
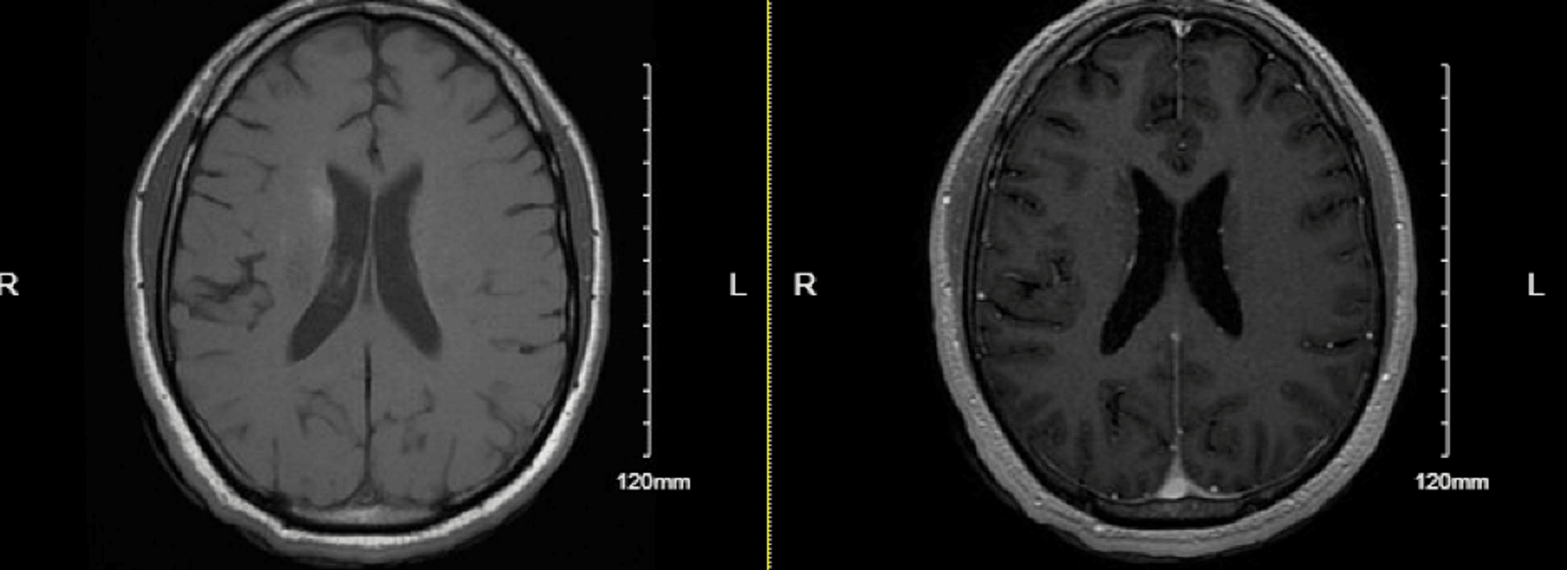
One-month post treatment MRI non-contrast Radiology impression includes interval improvement in areas of T2 prolongation, which have enhancement in the parenchyma and leptomeninges within the bilateral cerebellar hemispheres and right basal nuclei in comparison with initial MRI of the brain. Minimal residual enhancement remains. No midline shift or uncal herniation are present. No new areas of abnormal enhancement within the cerebral or cerebellar parenchyma are present.

## Discussion

*C. neoformans* is an encapsulated yeast that can be found in old pigeon droppings and can cause a spectrum of infections, ranging from asymptomatic airway colonization to severe disseminated disease or meningitis [7,8]. While immunocompromised individuals (e.g., HIV/AIDS patients with CD4 counts <100 cells/μL) are at highest risk, immunocompetent hosts may also develop progressive infections, particularly in the central nervous system [2,5]. Globally, cryptococcal meningitis causes an estimated 220,000 cases annually, with sub-Saharan Africa and Asia bearing the highest burden [4]. However, as seen in this case, sporadic infections occur even in seemingly healthy individuals. 

Cryptococcal meningitis typically presents as subacute meningoencephalitis, with symptoms such as headache, altered mental status, fever, neck stiffness, nausea, and vomiting [2]. In HIV-positive patients, symptoms may be milder or nonspecific, whereas immunocompetent individuals often experience prolonged symptom duration (6-12 weeks) [5]. Visual disturbances (e.g., diplopia, photophobia) or hearing loss may occur due to elevated intracranial pressure (ICP) or cranial nerve involvement [3]. The 49-year-old immunocompetent man in this case presented with a three-day history of throbbing headache, nausea, vomiting, vertigo, and transient sensory deficits, initially misdiagnosed as a stroke. His symptoms persisted despite treatment, and he later developed bilateral hearing loss, a finding consistent with cryptococcal meningitis-related cranial nerve involvement [3]. His lymphocytic pleocytosis, elevated CSF protein (70 mg/dL), low glucose (38 mg/dL) and opening pressure of 29 cm H_2_O aligned with classic cryptococcal meningitis findings [2]. Notably, his ICP rose to 36 cm H_2_O during treatment, mirroring data where >50% of cryptococcal meningitis patients had ICP >25 cm H_2_O [3].

*C. neoformans* reproduces by budding, forming round yeast cells with a polysaccharide capsule that evades immune detection [6]. Diagnosis relies on CSF analysis, antigen testing, and culture. In this case, CSF culture confirmed *C. neoformans*, with a positive antigen titer (1:32) consistent with active infection despite the patient’s normal CD4 count (641 cells/mm³). This aligns with rare reports of cryptococcal meningitis in immunocompetent hosts [4,5]. First-line therapy for cryptococcal meningitis combines liposomal amphotericin B (4 mg/kg/day) and flucytosine (25 mg/kg Q6H) for induction, followed by fluconazole consolidation [4,9]. This regimen was initiated here, with symptomatic improvement after ICP reduction via serial lumbar punctures, a critical intervention given his rising pressure [3]. The patient responded well to therapy, with resolution of headaches and improved imaging after one month. His antigen titer declined to 1:64, suggesting treatment efficacy. However, his lack of identifiable immunodeficiency underscores the importance of considering cryptococcal meningitis even in immunocompetent patients with subacute neurological symptoms [4,5].

Mortality in cryptococcal meningitis can be attributed to the increase in ICP. The mechanism of raised ICP is thought to be due to the fungi obstructing CSF reabsorption leading to communicating hydrocephalus and eventually leading to herniation and death if not treated [10]. This also explains the interstitial edema seen early in the case as the raised ICP and communicating hydrocephalus can result in fluid being pushed into the periventricular space, seen as a hyperintensity on MRI. Multiple lumbar punctures to reduce ICP have been shown to decrease mortality [10]. In immunocompromised patients, in particular, raised ICP can be caused by immune reconstitution inflammatory syndrome. During HIV immune reconstitution with concomitant infection, the immune system has an exaggerated response resulting in an average mortality of 20% in this population [11]. This only occurs in 25% of patients with HIV but marks an important consideration when giving treatment [11].

While cryptococcal meningitis is classically HIV associated, this case reflects the rising recognition of cryptococcosis in immunocompetent populations. The optimal treatment duration for non-HIV patients remains unclear due to limited data, highlighting the need for inclusive clinical trials [4]. This case of cryptococcal meningitis in an immunocompetent patient challenges conventional clinical paradigms and offers critical insights that expand upon current literature. The patient was initially misdiagnosed with a stroke due to nonspecific MRI findings, illustrating how cryptococcal meningitis’s neuroimaging can overlap with ischemic stroke or demyelinating processes, potentially delaying diagnosis [2]. Early CSF analysis remains essential to ensure timely recognition and treatment of cryptococcal meningitis across all immune phenotypes.

## Conclusions

This case demonstrates that CM should remain part of the differential diagnosis for subacute neurological symptoms even in immunocompetent individuals and when early imaging suggests alternative etiologies. Prompt CSF evaluation and timely initiation of antifungal therapy are critical for favorable outcomes. Clinicians should maintain a broad diagnostic perspective and consider fungal meningitis in patients with persistent or progressive neurological symptoms regardless of immune status. 
